# Spectroradiometric Determination of the Freezing Temperature of Gold

**DOI:** 10.6028/jres.095.006

**Published:** 1990

**Authors:** Klaus D. Mielenz, Robert D. Saunders, John B. Shumaker

**Affiliations:** National Institute of Standards and Technology, Gaithersburg, MD 20899

**Keywords:** blackbody, electrically calibrated detectors, integrating spheres, lasers, radiometry, silicon detectors, spectroradiometry, temperature

## Abstract

A direct spectroradiometric determination of the temperature of freezing gold was performed by measuring the spectral radiances of a gold blackbody relative to those of a laser-irradiated integrating sphere which was calibrated with absolute silicon detectors and an electrically calibrated radiometer. The measurements were performed at three laser wavelengths near 600 nm, and the temperature of the blackbody was calculated by substituting the measured spectral radiances into Planck’s radiation formula. The result obtained, *T*_Au_=(1337.33± 0.34) K, is 0.25 K below the gold-point assignment in the International Practical Temperature Scale of 1968 (IPTS-68) and has been adopted in September 1990 as the new gold-point value in the International Temperature Scale of 1990 (ITS-90). The effect of this change in the gold-point assignment on pyrometric, radiometric, and photometric measurement services provided by the National Institute of Standards and Technology is assessed.

## 1. Introduction

In this paper, we describe a new spectroradiometric measurement of the freezing temperature of gold based on absolute detector standards. Before describing our measurement, we review the definitions of this temperature in the International Practical Temperature Scale of 1968 (IPTS-68, [1]) and in the International Temperature Scale of 1990 (ITS-90, [2]), assess its significance for pyrometric, radiometric, and photometric measurement services provided by the National Institute of Standards and Technology, and review gold-point measurements performed elsewhere since the adoption of the IPTS-68.

The IPTS-68 allows the realization of temperatures, *T*_68_, above the freezing temperature of gold by means of the equation:

$$L_{\lambda }\left(T_{68}\right)/L_{\lambda }\left(T_{68,\text{Au}}\right)=P\left(\lambda ,T_{68}\right)/P\left(\lambda ,T_{68,\text{Au}}\right),$$ (1)

where

$$T_{68,\text{Au}}=1337.58K$$ (2)

is the temperature assigned to the gold point; *L*_λ_(*T*) is the spectral radiance [Wcm^−3^sr^−1^] at wavelength λ of a blackbody of temperature *T* [K];

$$P\left(\lambda ,T\right)=\left(c_{1}/n^{2}\lambda^{5}\right)/\left\{\exp\left[c_{2}/n\lambda T\right]-1\right\}$$ (3)

is the theoretical blackbody spectral radiance (Planck’s radiation formula) for light with air wavelength λ propagating in air with refractive index *n*; and *C*_1_ and *C*_2_ are the first and second radiation constants. The gold-point temperature given in eq (2) was derived from gas thermometry.

The new ITS-90 was adopted by the International Committee of Weights and Measures (CIPM) in September 1989 [2]. It supersedes the IPTS-68 and incorporates a new value for the freezing temperature of gold which is identical to the one reported in this paper [eq (35), below]. The ITS-90 extends the radiometric temperature definition of eq (1) down to the freezing point of silver, and allows the realization of radiation temperatures in terms of any one of the silver [*T*_90.Ag_= 1234.93 K], the gold [*T*_90.Au_= 1337.33 K], or the copper [*T*_90.Cu_= 1357.77 K] freezing points. The *T*_90_ values of the silver, gold, and copper points are believed to be self-consistent to such a degree that the substitution of any of them in place of the value of *T*_68.Au_ in eq (1) will not result in significant differences in the measured values of *T*_90_.

## 2. Radiometric Significance of the Gold Point

Realizations of the NIST scales of radiance temperature, spectral radiance, spectral irradiance, and photometric quantities, and the transfer of these scales to calibrated lamp standards, have been based on measurement chains derived from gold-point blackbodies for many years [3]…[6]. Although the instrumentation and procedures used have undergone refinements in the meantime, the IPTS-68 gold-point assignment has remained the starting point for these scale realizations for the past 2 decades. Consequently, the change of the gold-point temperature will affect all of these scales. The magnitude of the scale changes may be estimated as follows.

The realization of radiometric scales in terms of a primary gold-point standard involves measurements at specified wavelengths of the spectral radiance ratios of the gold-point blackbody and other blackbody sources which may then be used as secondary standards over wide temperature and wavelength ranges. The basic measurement equation for these radiance transfers is eq (1), which allows the determination of the temperature of a secondary standard source from the measured radiance ratio *L*_λ_(*T*)/*L*_λ_(*T*_AU_) and the assigned value of *T*_Au_. In the Wien approximation of Planck’s radiation formula,

$$P\left(\lambda ,T\right)∼\left(c_{1}/n^{2}\lambda^{5}\right)\;\exp\left(-c_{2}/n\;\lambda \;Τ\right),$$ (4)

we obtain

$$L_{\lambda }\left(T\right)/L_{\lambda }\left(T_{\mathrm{Au}}\right)∼\exp\left[\left(c_{2}/\lambda \right)\left(1/T_{\mathrm{Au}}-1/T\right)\right]$$ (5)

and, hence, by implicit differentiation with respect to *T* and *T*_Au_,

$$dT/T^{2}-dT_{\text{Au}}/T_{\text{Au}}^{2}∼0,$$ (6)

if the radiance ratio on the left-hand side of eq (5) has a fixed value. This shows that a radiance temperature *T* based on a gold-point value *T*_Au_ will be replaced by *T* + *∆T*, where

$$\Delta T∼{\left(T/T_{\text{Au}}\right)}^{2}\Delta T_{\text{Au}},$$ (7)

on a redefined scale in which *T*_Au_ is replaced by *T*_Au_+*∆T*_Au_.

When this result is substituted into the Wien approximation for the spectral radiance of the blackbody at temperature *T*, we obtain

$$\begin{array}{l} \Delta P\left(\lambda ,T\right)=\left[\partial P\left(\lambda ,T\right)/\partial T\right]\Delta T∼P\left(\lambda ,T\right)c_{2}\Delta T/\left(\lambda T^{2}\right)\\ \;∼P\left(\lambda ,T\right)c_{2}\Delta T_{\text{Au}}/\left(\lambda T_{\text{Au}}^{2}\right) \end{array}$$ (8)

Thus, the relative change in spectral radiance due to a change ∆*T*_Au_ in the gold point is

$$\begin{array}{l} \Delta P\left(\lambda ,T\right)/P\left(\lambda ,T\right)∼c_{2}\Delta T_{\text{Au}}/\left(\lambda T_{\text{Au}}^{2}\right)\\ \;=1.44\times 10^{-2}\Delta T_{\text{Au}}/\left(\lambda {Τ}_{\text{Au}}^{2}\right), \end{array}$$ (9)

where *T* is expressed in kelvins and λ in meters. This result is independent of the temperature of the blackbody and holds for spectral irradiance measurements as well, since radiance/irradiance transfers are based on purely geometrical considerations.

The NIST photometric scales (luminous intensity and luminous flux) are realized and maintained at *T*=2856 K (CIE Source A) by direct comparison with the NIST spectral irradiance scale. The effect of a reassignment of the gold point may be estimated by evaluating eq (9) at the Crova wavelength for Source A (λ=570 nm) [7]. This gives

$$\Delta Q_{v}/Q_{v}=1.41\times 10^{-2}\Delta T_{\text{Au}},$$ (10)

where ∆*T*_Au_ is again expressed in kelvins, and *Q*_v_ denotes a generalized photometric quantity.

From eqs (7), (9), and (10), it can now be estimated that the −0.25 K change in the IPTS-68/ITS-90 gold-point assignments will result in the approximate changes of reported calibration values listed in table 1. Although these changes are well within the quoted uncertainties for routine NIST measurement services, they will have an effect to reconcile small discrepancies between radiometric scales that have been observed in recent years. For example: In an intercomparison between independent radiometric scales based on silicon-photodiode physics, gold-point blackbody radiation, and synchrotron radiation, Schaefer, Saunders, and Hughey [8] found that the blackbody scale indicated a spectral irradiance at 600 nm which was about 0.8% higher than the detector scale, and about 1% higher than the synchrotron scale. The −0.25 K adjustment of the gold-point temperature improves the overall agreement of these intercomparisons.

In an international intercomparison of photometric base units, conducted by the Comite Consultatif de Photometrie et Radiometrie (CCPR) in 1985 [9,10], the NIST luminous intensity data were the only ones derived from a gold-point based measurement chain. They fell within the spread of the intercomparison, but were 0.5% higher than the average of the data reported by 14 other national laboratories, all of which had realized the candela with electrically calibrated radiometers. It may be seen from table 1 that the NIST data would have been within 0.1% of the world mean, had the gold-point temperature been 0.25 K lower.

## 3. Previous Radiometric Determinations of the Gold Point

The first measurement which projected a difference between the thermodynamic temperature of the gold point and its assigned IPTS-68 value was a precision determination of the Stefan-Boltzmann constant which was published by Blevin and Brown [11] of the Australian National Standards Laboratory (NSL) in 1971. Based on the IPTS-68 gold-point assignment their experiment, in which an electrically calibrated radiometer (ECR) was used to measure the spectrally total radiance of a blackbody radiator at the freezing temperature of gold, yielded a result which was about 0.1% lower than the value of the Stefan-Boltzmann constant (2π^5^/l5*h*^3^*c*^2^) calculated from the best values of the speed of light, *c*, and the Planck and Boltzmann constants, *h* and *k*. Blevin and Brown suggested that their experiment could be regarded as a radiometric determination of the gold point based on the theoretical value of the Stefan-Boltzmann constant, the corresponding result for the gold point being 1337.27 K, about 0.3 K lower than the IPTS-68 value.

Guildner and Edsinger [12] of the U.S. National Bureau of Standards noted that this result was consistent with their own work, which had revealed that sorption errors in gas thermometry caused a systematic difference between thermodynamic and IPTS-68 temperatures. Assuming that the effects of sorption were approximately linear with wavelength, they predicted that the temperature of freezing gold on the thermodynamic scale will prove to be lower than the value on the IPTS-68 by at least some 0.1 K. They suggested, however, that the IPTS-68 should not be changed without better understanding of the thermodynamic scale, as realized experimentally.

In 1975, Bonhoure [13] of the Bureau International des Poids et Mesures (BIPM) in France reported pyrometric measurements of the temperature differences of variable-temperature blackbodies in the 904 to 1338 K range, one kept at the IPTS-68 antimony temperature (903.89 K) and the other at selected higher temperatures. Assuming the antimony point to be correct, Bonhoure found that his measurements were in 0.05 K agreement with the IPTS-68 near the gold-point. Andrews and Gu [14] repeated this measurement at the National Physical Laboratory (NPL) of the U.K., using a photon-counting optical pyrometer to compare a gold-point blackbody to a reference source at the antimony point. They, too, concluded that the IPTS-68 gold-point value was not in error.

In the meantime, Guildner and Edsinger [15] had achieved significant improvements of high-temperature gas thermometry and showed that the thermodynamic temperatures from the triple point of water up to 730 K were consistently lower than the IPTS-68 values. Jung [16] of the German Physikalisch-Technische Bundesanstalt (PTB) carried this work further by performing pyrometric measurements at the antimony point relative to a blackbody at a thermodynamic temperature of 729.15 K, as established by gas thermometry. His work showed that the IPTS-68 antimony-point assignment was in fact too high by 0.15 K, thus necessitating numerical corrections to the gold-point values obtained by Bonhoure and by Andrews and Gu. The corrected values, as reported by Tischler and Rebagliati [17] and Hudson et al. [2], respectively, were 1337.20 and 1337.33 K. An expression equivalent to eq (7), above, was used to make the corrections.

Tischler and Rebagliati [17] also recalculated the results of five other researchers who had performed pyrometric measurements of the thermodynamic temperature difference between the silver and gold points between 1973 and 1982. To allow a uniform analysis, Tischler and Rebagliati interpreted all of these measurements as being performed with the silver point as the reference. The five gold-point values so recalculated ranged from 1337.18 to 1337.29 K, their average being 1337.22 K. The silver-point reference temperature assumed was 1234.88 K, a value which is only 0.01 K lower than more recent results obtained by Jones and Tapping [18] and Fischer and Jung [19], and 0.05 K lower then the ITS-90 value[2].

The data described above are summarized in table 2, with uncertainties quoted at the three standard-deviation level. The values given are all lower than the IPTS-68 gold-point assignment.

It should be noted, however, that the only direct determination of the gold point is that by Blevin and Brown [11], which involved absolute radiometric measurements and interpretation of the data obtained in terms of a fundamental law of physics. The others are pyrometric measurements of radiance ratios which relied, directly or indirectly, on the IPTS-68 assignment of the antimony point and agreed with the Blevin and Brown measurement only after allowance was made for an error in the antimony assignment. We conclude that radiometric and pyrometric temperature measurements at higher temperatures are, in themselves, reliable but may be encumbered with uncertainties if they are based on lengthy measurement chains which involve lower-temperature reference points. Our own measurement is similar to the Blevin and Brown experiment in that it is a direct measurement based on absolute radiometry, but differs from it in that we measured the spectral radiance of a gold-point blackbody and used Planck’s law to derive its temperature.

## 4. Apparatus

### 4.1 Overview

Our technique consisted of measuring the spectral radiance of a uniform, monochromatic source using two independent radiometric scales established by a standard silicon photodiode and an ECR. As the uniform, monochromatic source we used the exit port of an integrating sphere irradiated by monochromatic laser light near 600 nm. Then, by comparing the spectral radiance of this source to that of a gold-point blackbody with a monochromator-based spectroradiometer, we determined the blackbody radiance and calculated the gold-point temperature from Planck’s radiation formula. The use of independent detector standards provided redundancy of the measured data and increased the likelihood of high accuracy and internal consistency of the results.

**Figure 1:**
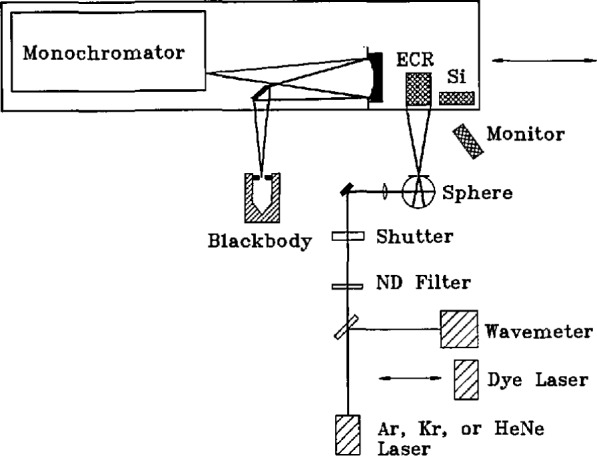
Gold-point measurement apparatus.

Figure 1 shows a diagram of the arrangement of the equipment used in these measurements. The three detector systems (silicon diode, ECR, and spectroradiometer) are mounted on a movable carriage which permits each of them to be moved into position to view the laser-irradiated integrating sphere. In addition, the carriage can be positioned so that the spectroradiometer views the gold-point blackbody.

Also indicated is a silicon monitor diode which is fixed above the sources and detectors and views the laser sphere from slightly outside the field of view of any of the other detectors. The purpose of this monitor was to compensate for small fluctuations of the laser-sphere source and for the presence of a neutral-density filter which was inserted in the beam for the silicon-diode and spectroradiometer measurements, as described in section 4.7.

Most of the simple operations of the equipment are computer controlled. This includes controlling the blackbody heater current, positioning the movable carriage, operating the spectroradiometer wavelength drive, triggering the simultaneous measurements by the monitor detector and the other detector systems, operating the shutter for dark-current measurements, and setting the integration time for the DVM readings (usually 5 s).

Throughout the discussion which follows, we state numerical estimates of potential sources of systematic error. These estimates are upper limits of systematic errors and will be combined in quadrature with the three-sigma random uncertainties of our measurements.

### 4.2 Gold-Point Blackbody

**Figure 2:**
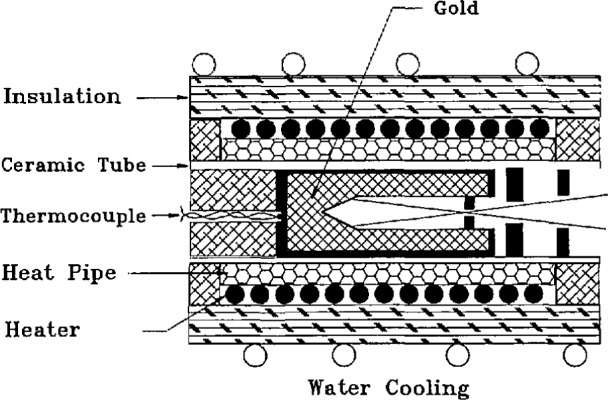
Cross section of heat-pipe blackbody furnace.

**Figure 3:**
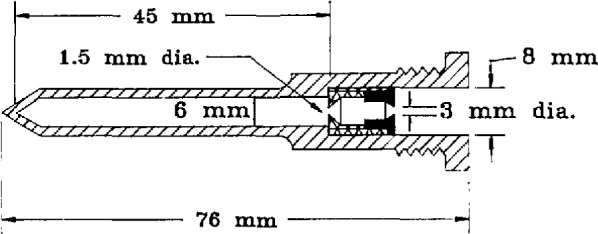
Blackbody inner cavity dimensions.

The blackbody construction is sketched in figures 2 and 3. The furnace incorporates a cylindrical heat pipe between the electrical heater and the alumina tube which holds the crucible of gold. The crucible and inner cavity are made of graphite, as are many of the rings which limit the viewing cone in front of the crucible and support the thermocouple tube behind the crucible. A slow flow of argon is directed from the back to the front of the furnace within the alumina tube, and Fittings on its ends permit maintaining a slight overpressure of argon except when the front shutter is opened for radiance measurements. The front graphite rings are regarded as sacrificial, and after five or ten melt-freeze cycles a few of the outermost rings had generally become partially consumed by oxidation and were replaced.

**Figure 4:**
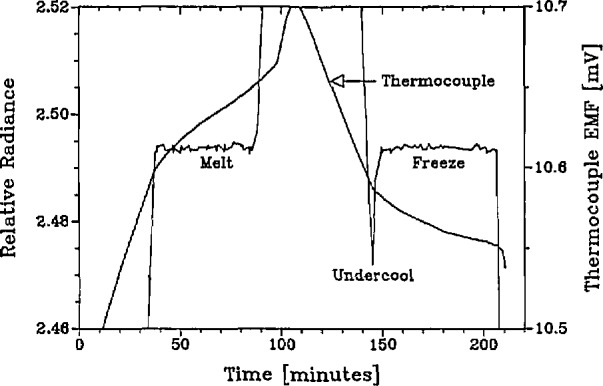
Spectroradiometric blackbody melt and freeze curve (514 nm).

A microcomputer controls the heater power supply by turning the furnace on, ramping the power up gradually over several hours, and finally alternating power levels to produce melts and freezes which typically last about 45 min each. The thermocouple provides the computer with the necessary continuous information about the state of the furnace which governs the succession of power changes. Spectroradiometric data from a representative melt-freeze cycle are shown in figure 4.

To confirm the radiometric adequacy of the blackbody design, several of the easily changed design parameters were tested by performing spectroradiometric matches between an ultra-stable vacuum tungsten strip lamp and the blackbody melts and freezes with modified blackbodies:

1. Furnaces with different internal diameters were compared. The two designs provided *f*/10 and *f*/6.5 viewing solid angles and crucibles containing 0.7 and 1.3 kg of gold, respectively.
2. Two different samples of nominally 0.99999 pure gold, acquired at different times, were compared in the smaller furnace, and a third sample in the larger furnace.
3. Three different inner blackbody cavity openings were compared. Their diameters were 1.0, 1.5, and 2.0 mm.

None of these changes affected the spectral radiance of the cavity within 0.01%. For the measurements reported here, the larger furnace with the crucible containing 1.3 kg of gold and 1.5-mm diameter aperture was used. For the calculation of the gold-point temperature from our spectral radiance measurements and the Planck function, we used an emissivity of 0.9999+0.0001, calculated from the dimensions of the cavity by Quinn’s and Ford’s technique [20,21], assuming uniform temperature.

**Figure 5:**
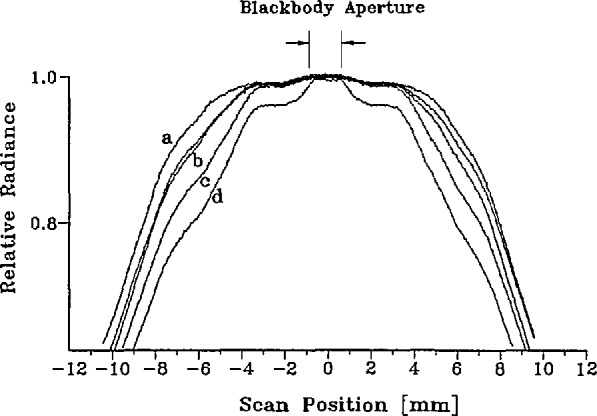
Relative spatial distribution of blackbody radiance (647 nm) at different times during melts and freezes. (a) end of melt (b) beginning and middle of melt (c) beginning of freeze (d) end of freeze.

Since heat flows from the outer heat pipe to the inner cavity during melts, and tends to flow from the inside to the outside during freezes, we expected differences in the relative spatial distributions of the blackbody radiance during melts and freezes, as well as changing distributions during the course of a melt or freeze. Relative radiance distributions were measured during melts and freezes carried out for this purpose, by moving the spectroradiometer in 0.6-mm steps across the face of the blackbody furnace, and by raising and lowering the furnace in 1.8-mm steps. Generally, four complete mappings could be acquired during the course of a single melt or freeze. We show in figure 5 examples corresponding to different times during a melt or freeze. In order to eliminate the effect of the change in the distribution during the 10 or 15 min required for each mapping, at each point of the source plane the four spectroradiometer readings obtained during the complete melt or freeze were fitted to a linear function of the time of measurement. Then, “instantaneous” mappings were constructed by interpolation and used in the computation of the spectroradiometer spatial apparatus function correction *f*_H_ defined in section 5.2.3.

### 4.3 Laser Sphere

The laser-sphere source is a 50-mm diameter copper integrating sphere with a 6-mm diameter entrance port located about 120° from a 20-mm diameter exit port. The interior of the sphere is coated with a 3-mm thick layer of compacted Halon (polytetrafluoroethylene) powder [22]. On the outside of the sphere, a single loop of copper cooling water tubing is soldered to each hemisphere.

A 20-W argon ion laser, a 5-mW helium-neon laser, and a 6-W krypton ion laser were used to illuminate the sphere at 514, 633, and 647 nm. The air wavelengths assigned to these laser transitions in Ref. [23] are given in table 3. The power output of the lasers remained stable to better than 1% over several hours, with no further stabilization than the built-in standard power monitoring system of the laser. Since all of our measurements on the laser-sphere were made relative to a simultaneously recorded signal from the monitor detector, this stability was adequate. The helium-neon laser provided too little power for accurate ECR readings, so at 633 nm we only compared silicon-detector and spectroradiometer measurements. The laser beams were directed by a series of plane mirrors through a quarter-wave plate to a lens which focused the beam to a point in the sphere entrance port.

Because the spectroradiometer is sensitive to linear polarization, it was necessary to know the polarization of all sources. The blackbody can safely be assumed to be unpolarized, but we felt less certain about the laser-sphere source since the laser beam is so highly linearly polarized. Consequently, we inserted the quarter-wave plate in the laser beam to convert its linear polarization to circular polarization entering the sphere. Then we measured the linear polarization of the laser-sphere source by the use of a rotating sheet polarizer and, as a polarization indifferent detector, another integrating sphere with a silicon detector in its exit port. With careful attention to alignment, this technique yielded consistent values of 0.0007+0.0003 for the degree of linear polarization of the sphere output. This is sufficiently small, so that we treated the laser-sphere source as unpolarized. We included in our error analysis a polarization uncertainty of ±0.02%, which is the product of the measured degree of polarization of the sphere output and the polarizance of the spectroradiometer (0.3) in the spectral region of interest.

**Figure 6:**
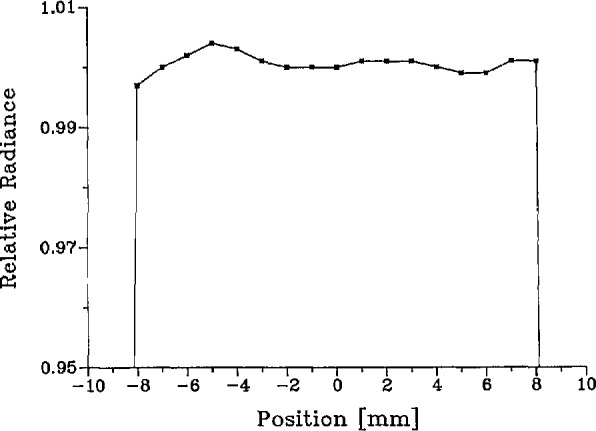
Relative horizontal distribution of laser sphere radiance at exit aperture (514 nm).

The relative spatial radiance distribution of the laser-sphere source was measured with the spectroradiometer. The exit aperture of the sphere was imaged with a tiltable spherical mirror into the source focal plane of the spectroradiometer. Then, the horizontal scanning capability of the spectroradiometer, coupled with the vertical tilting motion provided by the mirror, permitted a complete mapping of the 255-mm^2^ source area in a 1×1 mm grid. Initial attempts to perform this mapping showed a fine structure which depended strongly upon laser beam positioning in the entrance port. This proved to be caused by a fine speckle pattern in the sphere output which we were able to smooth out by rotating one of the plane mirrors to introduce a small, rapid wobble of the beam during the measurements. The spatial distributions thus obtained are reproducible, smooth, and independent of laser alignment and wavelength. In figure 6, we show the distribution at 514 nm.

Since the spectroradiometer views the laser sphere within a larger solid angle (approximately *f*/12) than either the silicon diode (*f*/19) or the ECR (*f*/25), we required that the laser-sphere source be Lambertian. We tested for uniformity of the irradiance field in the detector plane by translating the silicon-diode detector horizontally and vertically in the detector plane and recording the ratio of the signal obtained to that of the monitor detector. The cos^4^θ dependence expected for a Lambertian source was obtained to within +0.01% over our largest (*f*/12) viewing angle.

### 4.4 Spectroradiometer

The spectroradiometer used in our measurements was a Cary-14 prism-grating double monochromator^1^. The fore optics indicated in figure 1 consisted of a plane mirror and a 1-m radius-of-curvature spherical mirror. These were positioned to produce 1:1 images of sources on the entrance slit, while keeping the optical axis within less than 3° of the axis of the spherical mirror. A circular aperture stop at the spherical mirror limited the angular field of view of the radiometer to *f*/12 (except in the horizontal direction, in which it was limited to *f*/16 by the internal optics of the monochromator). A polished metal mask containing a 1.0-mm wide by 0.8-mm high rectangular opening was mounted just in front of the entrance slit. Since the slit width used for all measurements was 0.6 mm, the effective field stop of the spectroradiometer system was a rectangle 0.6-mm wide by 0.8-mm high. A telescope focused on the entrance slit mask gave the operator a magnified view of the source image at the slit, to help in the positioning and focusing of sources. In the detector compartment behind the exit slit of the monochromator, a photomultiplier and a small HeNe laser were mounted on an externally positionable sliding platform, so that either can be placed on axis. The photomultiplier is a thermoelectrically cooled, magnetically shielded, end-on, eleven-stage EMI 9658 tube with S-20 response. The laser is used to define the optic axis of the spectroradiometer system for the positioning and orienting of sources.

**Figure 7:**
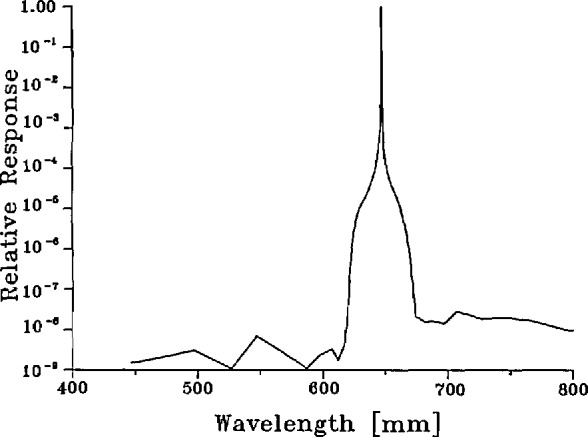
Slit-scattering function of spectroradiometer (647 nm).

Measurements of the spectroradiometer slit-scattering function have been described previously [24], They employed a laser-sphere source and consisted of measurements over a broad range of dye and ion laser wavelengths while the monochromator remained set at a fixed wavelength. These measurements were supplemented by information obtained by scanning the monochromator wavelength setting while viewing a constant ion laser line. An example of the resultant slit-scattering function is shown in figure 7. Using these slit-scattering functions and the definition of eq (31), below, we can calculate the spectral slit width. During the course of the measurements reported here, the slit-scattering function was measured at each wavelength by performing abbreviated monochromator scans which covered only about 10 nm. Consequently, the spectral slit widths calculated from these data were corrected for the truncation of the far wings by multiplying by a factor 1.0025+0.0004, obtained from the earlier, complete scans by similarly truncating those data. For our nominally 0.6-mm slit widths, we obtained bandwidths in the neighborhood of 2.5 nm. The factor σ(λ,λ_L_) in eq (31) causes the slit width to depend on the precise coincidence of the monochromator wavelength, λ, at the laser wavelength, λ_L_. The uncertainty of this coincidence (+0.05 nm) is included in the composite random uncertainty of our measurements, as discussed in section 6.6.

**Figure 8:**
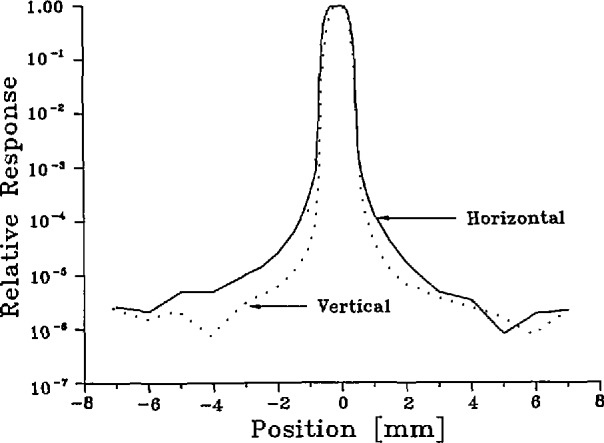
Spatial apparatus function of spectroradiometer (514 nm).

To measure the spatial apparatus function of the spectroradiometer, we aimed it at the center of the laser-sphere source and mounted a mask containing a 0.3-mm diameter pinhole in the focal plane just in front of that source. Then we recorded the spectroradiometer signal as the mask was translated horizontally and vertically across the source aperture. These traverses were then separately normalized to unity at the center. Examples are shown in figure 8. We found the spatial apparatus function to be essentially independent of wavelength over the spectral range used in our experiment.

**Figure 9:**
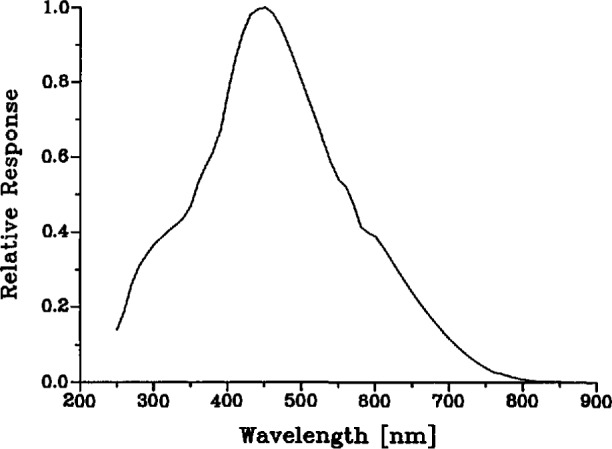
Relative spectral response of spectroradiometer.

Although our gold-point measurement does not rely on an absolute calibration of the spectroradiometer, the spectral responsivity *R*_s_(λ) of the instrument is needed to evaluate the slit-function correction of the measured blackbody radiance [eq (32), below]. We determined *R*_s_(λ) with an accuracy sufficient for this purpose by calibrating the spectroradiometer with a standard tungsten-strip lamp. The result of this calibration is shown in figure 9.

### 4.5 Silicon Diode

**Figure 10:**
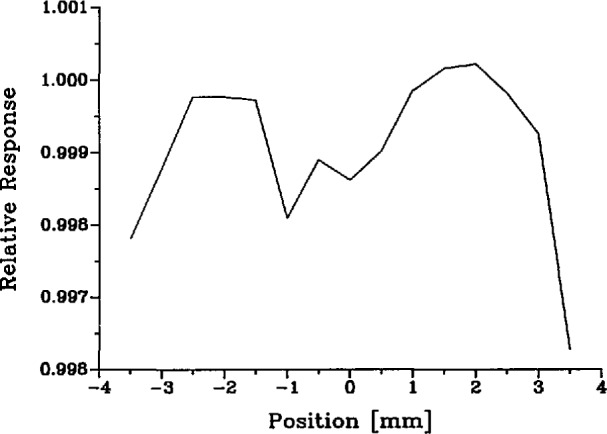
Relative horizontal distribution of silicon-diode responsivity (633 nm).

The silicon diode detector is a windowless, 1-cm^2^, p-n type, Hamamatsu 1337-1010Q diode operated without temperature control. The response of this detector type has been found to be quite independent of temperature at ambient temperatures in the 500 to 700 nm spectral region [25]. The spatial uniformity of the diode response was mapped over a 7×7 mm grid with a 633-nm laser beam and was found to be better than ±0.1%, as shown in figure 10.

The silicon diode was calibrated by senior staff of the NIST Photodetector Metrology Project with a stated three-standard-deviation uncertainty of ±0.2%. This calibration was performed using a monochromator-based spectral response comparator and a high-accuracy, laser-based detector characterization facility. The diode spectral responsivities at 514, 633, and 647 nm were measured by comparison to three fully characterized, absolute QED-200 detector standards. The QED standard is a light-trap configuration of n-p type, UDT UV-100 silicon diodes which, by significantly reducing reflection losses, permits a simplified, accurate self-calibration procedure [26]. During our measurements, we observed no changes in the diode response with respect to the ECR and monitor-diode readings. The overall agreement of the silicon-diode and ECR readings taken at 514 and 647 nm was on the order of 0.05% (see table 7).

### 4.6 Electrically-Calibrated Radiometer

**Figure 11:**
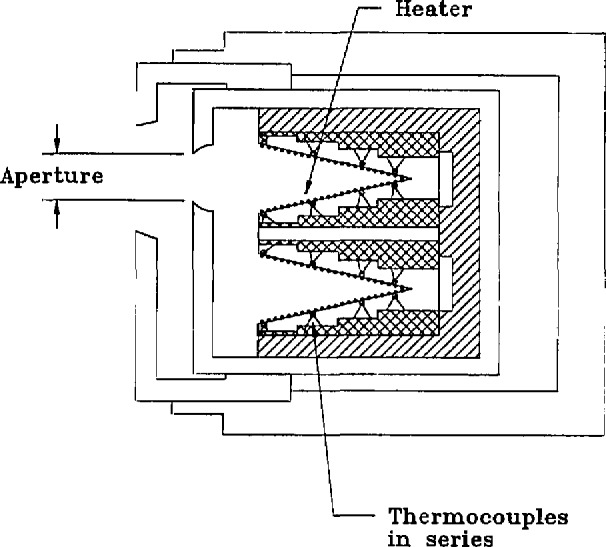
Cross Section of ECR.

**Figure 12:**
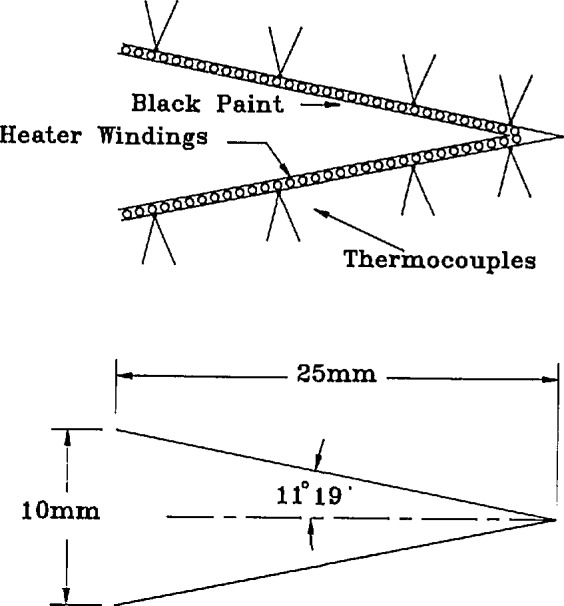
ECR cone details.

The ECR used was built by the National Institute of Measurement and Testing Technology (NIMTT) of the P.R.C. and is similar to that described in reference [27]. As shown in figures 11 and 12, it has two identical conical copper receivers of 11° 19′ half angle, 25-mm length, and 10-mm opening. The heater windings are made of 0.03 mm diameter manganin wire, are uniformly and tightly glued to the insides of the cone, and are coated with black paint and kerosene smoke. The outside of each cone is equipped with a uniform, circular array of 72 nickel-chromium, constantan (type “E”) thermocouples made of 0.1-mm wire. Only one cone is exposed to radiant or electrical heating, respectively, and the thermocouple arrays on the two cones are connected oppositely. In this manner, only the temperature difference of the cones is registered and environmental temperature changes (such as those caused by adiabatic air pressure fluctuations [28]) are largely canceled. The laser-sphere aperture and its distance from the ECR were chosen so that the irradiated and electrically heated portions of the ECR cone were closely matched.

Due to residual differences in the electrical and radiant heating of an ECR, the temperature rise produced by radiant power is not exactly the same as that produced by an equal amount of electrical power. This non-equivalence of electrical and radiant heating has been examined in detail by Hengstberger [29]. The determination of the small correction for this non-equivalence is generally a one-time characterization of the ECR, as contrasted with the routine calibration by electrical substitution heating to achieve a thermocouple signal which matches that of an unknown radiant input power. Our characterization of the ECR was based on the equations

$$S_{\mathrm{ECR}}=R_{\mathrm{ECR}}P^{\prime },$$ (11)

$$P^{\prime }=P\left(1+\kappa -\rho -\eta -\epsilon \right),$$ (12)

where *S*_ECR_ is the ECR signal (thermocouple EMF), *R*_ECR_ is its responsivity, *P′* is the thermal power at the thermocouple hot junctions, *P* is the total (radiant or electrical) power applied to the ECR, and the remaining quantities are small correction terms:

κ is the environmental heating correction,
ρ is the reflection/emission loss,
η is the air convection/conduction loss,
∊ is the sum of other losses.

The last term, *ϵ*, denotes the sum of all losses (such as thermal emission from the back side of the cone) which are identical for radiant and electrical heating and, therefore, do not require a correction. The other correction terms depend on the type of heating and will accordingly be distinguished by subscripts, r and e, so that *κ*_r_ denotes radiant case heating, *κ*_e_ denotes electrical lead heating, and so on. The characterization of the instrument consists of measuring the six correction terms *κ*_r_ *κ*_e_, *ρ*_r_, *ρ*_e_, η_r_, and η_e_ one at a time in specially designed experiments. To measure *κ*_r_ we covered the ECR aperture with a styrofoam plug and measured the thermopile signals with and without a radiant power *P*_r_ falling onto the face of the instrument. The lead heating effect was determined by rearranging the ECR circuitry so that the heat generated by the leads alone could be measured in addition to the heat generated by the heater windings and leads. The reflection/emission loss was measured by irradiating the ECR with a laser beam entering through the small central hole in a spherical mirror which could be rotated to focus the radiation remitted by the ECR back into the receiver cone, or to the side of it. The air convection/conduction correction terms were measured by radiantly and electrically heating the ECR in air and vacuum. These experiments have been described by Geist [30], The only correction which was measurably non-zero was the air convection/conduction term. It amounted to 0.1%.

**Figure 13:**
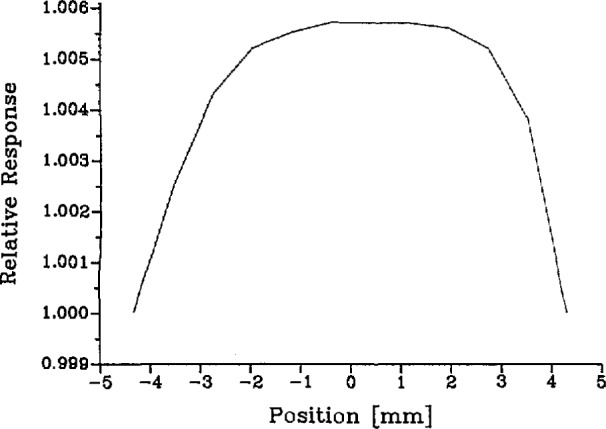
Relative horizontal distribution of ECR responsivity (514 nm).

To estimate the error caused by spatial non-uniformity of response, we scanned the ECR aperture with a 1-mm diameter, 514-nm laser beam, and also mapped the radiation field produced by the laser sphere at this aperture. The former was found to vary by 0.6% (see fig. 13), and the latter by +0.01%. The corresponding correction factor was evaluated as 0.9998+0.0005.

Since the ECR is presumed to be sensitive in the infrared, it was necessary to determine the contribution which heating of the laser-sphere source makes to the ECR signal. This contribution was estimated by measuring the radiation temperature rise of the sphere interior during laser irradiation at 514 nm, using an imaging infrared radiometer. The measured temperature rise was 0.09 K. Hence, the increment in the radiant power received by the ECR was calculated as 0.056 *μ*W, about 0.01% of the 0.4-mW radiant power levels used in our experiment.

The results of these various measurements and their estimated, three-sigma uncertainties are listed in table 4. We applied a 0.1% correction to the ECR responsivities measured by electrical substitution heating and estimated the overall systematic uncertainty of the ECR as 0.2%.

### 4.7 Monitor Diode and Neutral Density Filter

Any measurement of the laser sphere by any of the three detector systems was accompanied by a simultaneous measurement by the monitor detector, followed by dark-current readings obtained by blocking the laser input to the sphere. The ratio of the dark-current corrected detector signal to the dark-current corrected monitor signal was the quantity actually used in the subsequent analysis. As mentioned earlier, this suppressed errors due to fluctuations of laser-sphere output. The monitor diode was an EG&G 444B silicon photodiode. Its response was found to be stable within 0.01% over periods of several hours.

Because the optimal power for ECR measurements is about four orders of magnitude higher than the power level produced by the blackbody, a neutral density filter was inserted in the laser beam when silicon-diode and spectroradiometer readings were taken. The monitor-diode signals obtained with and without the filter provided measurements of the transmittance of this filter, so that our data were automatically adjusted for its presence when ratios were computed.

### 4.8 Detector Linearity

The linearity of response of the spectroradiometer and silicon detectors was measured with an automated beam conjoiner [31], a flux-addition device. For the range of signal levels and the operating conditions used in these gold-point measurements, we found no correction necessary for departures from strict linearity. The ECR was tested by varying the electric heater power and exhibited no detectable nonlinearity over the small dynamic range in which this instrument was used. We assessed the combined uncertainty due to detector nonlinearities to be less than 0.1%.

### 4.9 Apertures

**Figure 14:**
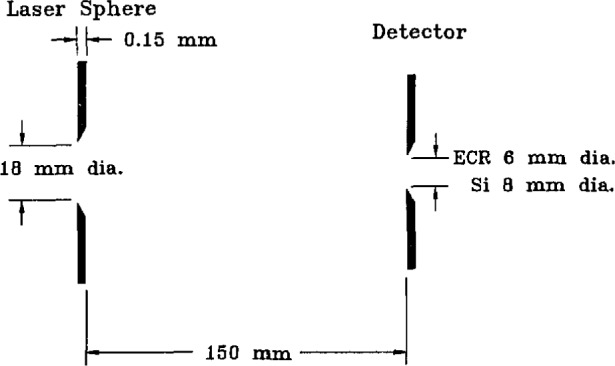
Laser-sphere and detector apertures.

The mutual arrangement of the laser-sphere and detector apertures is shown in figure 14. The laser-sphere aperture was oriented perpendicular to the spectroradiometer optic axis by turning on the alignment laser in the spectroradiometer, holding a glass microscope slide against the aperture, and adjusting the sphere until the laser beam was reflected by the glass back along itself. In a similar manner, the detector apertures were oriented using an auxiliary HeNe laser mounted on the source table but aimed into the spectroradiometer precisely along the spectroradiometer alignment laser beam. Because of the precision of these alignments, we believe that any error arising from non-parallelism of the apertures is negligible. These co-aligned lasers also provided means of positioning the centers of the detector and sphere apertures in the same horizontal plane, and of determining the spectroradiometer carriage positions at which the detectors are opposite the laser-sphere.

Reflections from the diode surface and from the diode and ECR apertures and mounting hardware could potentially be returned by the sphere aperture or hardware back to the detectors, or be scattered within the sphere and partially returned to the detectors. Although numerical estimates suggested that the errors due to such reflections are exceedingly small, two verification experiments were carried out. In the first, the detectors were covered by black velvet cloth except for the apertures and a narrow sliver at their edges. No detectable (<0.01%) signal differences were observed. In the second, the monitor signal was followed as the detectors were moved into position and away. Again, there was no detectable signal change.

## 5. Theory

### 5.1 Simplified Derivation of Measurement Equation

The basic principle of our measurement is best explained by temporarily neglecting small effects arising from the finite bandwidth of the sources and detectors involved, as well as all further effects introduced by their different sizes and spatial radiance distributions. With these simplifications, the spectroradiometer signals obtained at any given wavelength with the gold-point blackbody and laser-sphere sources are given by

$$S_{S}^{\mathrm{BB}}=L_{\lambda }^{\mathrm{BB}}\;\Delta \lambda \;G_{S}\;R_{S},$$ (13)

$$S_{S}^{\mathrm{LS}}=L^{\mathrm{LS}}\;G_{S}\;R_{S},$$ (14)

where $L_{\lambda }^{\mathrm{BB}}$ ∆λ and *L*^LS^, respectively, are the radiances of the blackbody and the laser sphere, ∆λ is the spectral bandwidth of the spectroradiometer, and *G*_s_ and *R*_s_, respectively, denote the geometric extent and the spectral responsivity of the spectroradiometer system. By taking the ratio of eqs (13) and (14), we obtain

$$L_{\lambda }^{\text{BB}}\Delta \lambda =\left(S_{S}^{\text{BB}}/S_{S}^{\text{LS}}\right)L^{\text{LS}},$$ (15)

there by eliminating the unknown properties of the spectroradiometer and relying only on the linearity of its response to ensure that this result is accurate. Similar to eq (14), the signal obtained with the silicon-diode or ECR reference detector and the laser sphere is

$$S_{D}^{\mathrm{LS}}=L^{\mathrm{LS}}\;G_{D}\;R_{D},$$ (16)

where *R*_D_ is the spectral responsivity of the detector, and where the geometric extent *G*_D_ is given by the standard expression which describes the radiation transfer between circular, parallel, and coaxial apertures with radii *r*_LS_ and *r*_D_, and separation *d* [32],

$$\begin{array}{l} G_{D}=\left(\pi^{2}/2\right)\{\left(d^{2}+r_{\mathrm{LS}}^{2}+r_{D}^{2}\right)-[{\left(d^{2}+r_{\mathrm{LS}}^{2}+r_{D}^{2}\right)}^{2}\\ \;{-4r_{\mathrm{LS}}^{2}r_{D}^{2}]}^{1/2}\} \end{array}$$ (17)

Combining eqs (15) and (16), we obtain the following approximate result for the spectral radiance of the blackbody in terms of the measured spectroradiometer and reference detector signals, the geometrical parameters of the laser sphere and reference detector arrangement, and the known spectral responsivity of the reference detector,

$$L_{\lambda ,0}^{\mathrm{BB}}=\left[S_{S}^{\mathrm{BB}}S_{D}^{\mathrm{LS}}/\left(S_{S}^{\mathrm{LS}}\;\Delta \lambda \;R_{D}\;G_{D}\right)\right].$$ (18)

The subscript (0) has been affixed to this expression for $L_{\lambda }^{\mathrm{BB}}$ in order to indicate that it is only an approximation to the full measurement equation. The corrections which must be applied to it for the rigorous reduction of measured data analysis will be derived next.

### 5.2 Derivation of Correction Factors

#### 5.2.1 Spectroradiometer Signals

The rigorous measurement equation describing the spectroradiometer signals obtained when the centers of the blackbody and laser-sphere source are imaged with unit magnification upon the small rectangular entrance slit of the instrument is [33]:

$$\begin{array}{l} S_{S}\left(\lambda \right)=∭L_{\lambda }\left(\lambda^{\prime },x,y\right)R_{S}\left(\lambda^{\prime }\right)\sigma \left(\lambda ,\lambda^{\prime }\right)d\lambda^{\prime }\\ \;\cdot H\left(x,y\right)dx\;dy. \end{array}$$ (19)

Here,

*S*_S_(λ) is the spectroradiometer signal;
λ is the wavelength setting of the monochromator and λ′ is an arbitrary wavelength;
*x* and *y* are cartesian coordinates in the source plane, referred to the source center (0,0);
*L*_λ_(λ′,*x*,*y*) is the spectral radiance of the source;
*R*_s_(λ′) σ(λ,λ′) is the spectral response of the spectroradiometer to radiation of wavelength λ′ when the monochromator is set at λ;
*H*(*x*,*y*) is the relative spatial response of the spectroradiometer to radiation from a source point (*x*, *y*) when the instrument is focused at the source center.

The integration in eq (19) and later expressions extends over all wavelengths and over the entire source aperture plane.

The factorization of the spectral response of the instrument into the two factors *R*_s_ and σ is uniquely defined if we require that R_s_(λ′) is independent of λ and that the maximum value of cr(λ,λ′) is exactly unity. Then σ(λ,λ′) is what is commonly known as the slit function shown in figure 7 (or, better, the slit-scattering function to emphasize the fact that the distant wings are due to diffraction and scattering), and R_s_(λ′) is the spectral responsivity of the instrument shown in figure 9, which is dominated by the spectral transmittance of the monochromator and the spectral response of the detector. With perfect imagery, the spatial apparatus function *H*(*x*,*y*) would be a constant everywhere within a 0.6×0.8-mm rectangle and zero elsewhere. However, due to diffraction and scattering from dust and optical imperfections we must allow for the departures from this ideal illustrated by figure 8. We assumed that, like the slit-scattering function, *H*(*x*,*y*) is normalized so that its maximum value is one, although this is not necessary since any such normalization factors will eventually cancel out in our application.

We replace the blackbody spectral radiance by a product of its value at the center and a relative spatial distribution function. The former is expected to be a Planckian function of wavelength. The latter is shown in figure 5. It exhibits a value close to unity over a small (1.5-mm diameter) central circular area, and then decreases to near zero at a radial distance of about 20 mm. Accordingly, we assume

$$L_{\lambda }\left(\lambda^{\prime },x,y\right)=L_{\lambda }^{\mathrm{BB}}\left(\lambda^{\prime }\right)\ell_{\mathrm{BB}}\left(x,y\right),$$ (20)

where *ℓ*_BB_(0,0)=1. This factorization of the spectral radiance into separate spectral and spatial factors is an acceptable approximation for the purposes of the integrals in eq (19) because the slit-scattering and spatial apparatus functions are both sharply peaked functions. Thus, the error in this approximation at large distances from the source center or at wavelengths far from the monochromator setting will make a negligible contribution to the value of the integrals. With this approximation to the spectral radiance, we can write the measurement equation for the spectroradiometer viewing the center of the blackbody source as

$$\begin{array}{c} S_{S}^{\mathrm{BB}}\left(\lambda \right)=\int L_{\lambda }^{\mathrm{BB}}\left(\lambda^{\prime }\right)R_{S}\left(\lambda^{\prime }\right)\sigma \left(\lambda ,\lambda^{\prime }\right)d\lambda^{\prime }\\ \cdot \iint \ell_{\mathrm{BB}}\left(x,y\right)H\left(x,y\right)dx\;dy. \end{array}$$ (21)

We also assume that the spectral radiance of the blackbody is Planckian, so that

$$L_{\lambda }^{\mathrm{BB}}\left(\lambda^{\prime }\right)=\frac{L_{\lambda }^{\mathrm{BB}}\left(\lambda_{L}\right)}{P\left(\lambda_{L},T\right)}P\left(\lambda^{\prime },Τ\right),$$ (22)

where λ_L_ is the wavelength of the laser-sphere source, *T* is the blackbody temperature, and *P*(λ,T) is the Planck function defined by eq (3). Thus we rewrite eq (21) as

$$\begin{array}{c} S_{S}^{\mathrm{BB}}\left(\lambda \right)=\frac{L_{\lambda }^{\mathrm{BB}}\left(\lambda_{L}\right)}{P\left(\lambda_{L},T\right)}\int P\left(\lambda^{\prime },Τ\right)R_{S}\left(\lambda^{\prime }\right)\sigma \left(\lambda ,\lambda^{\prime }\right)d\lambda^{\prime }\\ \cdot \iint \ell_{\mathrm{BB}}\left(x,y\right)H\left(x,y\right)dx\;dy. \end{array}$$ (23)

In order to estimate the possible error introduced by the approximations of eqs (20) and (22), we modeled the distributions involved by simple analytical functions which permitted the exact evaluation of the integrals in terms of elementary functions. By varying the parameters of these models to correspond in varying degrees to the measured functions, and observing the effect of the product approximation on eq (20), we found that the error is on the order of 0.002%.

We approximate the laser-sphere radiance in eq (19) by

$$L_{\lambda }\left(\lambda^{\prime },x,y\right)=\delta \left(\lambda^{\prime }-\lambda_{L}\right)L^{\mathrm{LS}}\left(0,0\right)\ell_{\mathrm{LS}}\left(x,y\right),$$ (24)

where the delta function reflects the fact that the spectral distribution is that of a sharp laser line at wavelength λ_L_, and L^LS^(0,0) is the central radiance of the source. The remaining factor *ℓ*_LS_(*x*,*y*) is the relative spatial radiance distribution of the source shown in figure 6, which is approximately unity over the circular exit aperture of the sphere and zero outside this aperture. By performing the integration over wavelength in eq (19), we obtain

$$\begin{array}{c} S_{S}^{\mathrm{LS}}\left(\lambda \right)=R_{S}\left(\lambda_{L}\right)\sigma \left(\lambda ,\lambda_{L}\right)L^{\mathrm{LS}}\left(0,0\right)\\ \cdot \iint \ell_{\mathrm{LS}}\left(x,y\right)H\left(x,y\right)dx\;dy. \end{array}$$ (25)

#### 5.2.2 Absolute Detector Signals

The rigorous measurement equation for the absolute (silicon-diode or ECR) detector signals is [33]

$$\begin{array}{c} S_{D}^{\mathrm{LS}}=\int \int \int \int \int L_{\lambda }\left(\lambda^{\prime },x,y\right)R_{D}\left(\lambda^{\prime }\right)d\lambda^{\prime }\\ \;\cdot \frac{\cos\theta \cdot \cos\theta^{\prime }}{d^{2}+{\left(x-x^{\prime }\right)}^{2}+{\left(y-y^{\prime }\right)}^{2}}dx\;dy\;dx^{\prime }\;dy^{\prime }. \end{array}$$ (26)

In this expression,

$S_{D}^{\mathrm{LS}}$ is the detector signal;
(*x*,*y*) and (*x*′,*y*′) refer to parallel cartesian coordinate axes in the source and detector aperture planes, respectively;
*θ* and *θ*′ are the angles between the normals to these aperture planes and the ray defined by the points (*x*,*y*) and (*x*′,*y*′);
*d* is the distance between the aperture planes;
*L*_λ_(λ′,*x*,*y*) is the spectral radiance of the laser-sphere source, assumed directionally uniform (Lambertian) within the small solid angle sampled by the detectors (*f*/19 for the silicon detector and *f*/25 for the ECR);
*R*_D_(λ′) is the spectral responsivity of the detector.

We assume again that the spectral radiance of the laser-sphere source is given by eq (24). We further assume that in the integrals of eq (26) the relative spatial distribution function can be replaced by its average value,

$$\langle \ell_{\mathrm{LS}}\rangle =\frac{1}{A_{\mathrm{LS}}}\cdot \iint \ell_{\mathrm{LS}}\left(x,y\right)dx\;dy,$$ (27)

and moved outside the integrals. *A*_LS_ is the laser-sphere aperture area. Thus eq (26) becomes:

$$\begin{array}{c} S_{D}^{\mathrm{LS}}=R_{D}\left(\lambda_{L}\right)L^{\mathrm{LS}}\left(0,0\right)\langle \ell_{\mathrm{LS}}\rangle \\ \cdot \int \int \int \int \frac{\cos\theta \cdot \cos\theta^{\prime }}{d^{2}+{\left(x-x^{\prime }\right)}^{2}+{\left(y-y^{\prime }\right)}^{2}}dx\;dy\;dx^{\prime }\;dy^{\prime }. \end{array}$$ (28)

For the geometry we used, this integral can be evaluated to give

$$S_{D}^{\mathrm{LS}}=R_{D}\left(\lambda_{L}\right)L^{\mathrm{LS}}\left(0,0\right)\langle \ell_{\mathrm{LS}}\rangle G_{D},$$ (29)

where *G*_D_ is the geometric extent previously defined by eq (17).

#### 5.2.3 Definition of Correction Factors

We can now combine eqs (23), (25), and (29) in order to derive the rigorous analog of the approximate expression (18) for the blackbody spectral radiance. This is accomplished by dividing eq (25) into eq (23), solving for $L_{\lambda }^{\mathrm{BB}}\left(\lambda_{L}\right),$ substituting the result for *L*^LS^(0,0) obtained from eq (29), and then multiplying the numerator and denominator by the spectral bandwidth ∆λ_L_ of the spectroradiometer at λ_L_. Thus we obtain

$$L_{\lambda }^{\mathrm{BB}}\left(\lambda_{L}\right)=\frac{S_{S}^{\mathrm{BB}}\left(\lambda \right)S_{D}^{\mathrm{LS}}\left(\lambda_{L}\right)}{S_{S}^{\mathrm{LS}}\left(\lambda \right)\Delta \lambda_{L}R_{D}\left(\lambda_{L}\right)G_{D}}\cdot \frac{f_{\sigma }f_{H}}{\langle \ell_{\mathrm{LS}}\rangle },$$ (30)

where the bandwidth ∆λ_L_ is defined as the normalized area of the slit-scattering function,

$$\Delta \lambda_{L}=\frac{\int \sigma \left(\lambda ,\lambda^{\prime }\right)d\lambda^{\prime }}{\sigma \left(\lambda ,\lambda_{L}\right)}.$$ (31)

The left-hand factor in eq (30) is identical to the approximate radiance of eq (18), and the right-hand factor is comprised of three correction terms:

1. A spectroradiometer slit-scattering function correction, defined as
  $$f_{\sigma }=\frac{P\left(\lambda_{L},T\right)R_{S}\left(\lambda_{L}\right)\int \sigma \left(\lambda ,\lambda^{\prime }\right)d\lambda^{\prime }}{\int P\left(\lambda^{\prime },T\right)R_{S}\left(\lambda^{\prime }\right)\sigma \left(\lambda ,\lambda^{\prime }\right)d\lambda^{\prime }}.$$ (32)
  This factor arises from the different spectroradiometer response to broadband blackbody radiation and monochromatic laser light.
2. A spatial apparatus function correction, defined as
  $$f_{H}=\frac{\iint \ell_{\text{LS}}\left(x,y\right)H\left(x,y\right)dx\;dy}{\iint \ell_{\text{BB}}\left(x,y\right)H\left(x,y\right)dx\;dy}.$$ (33)
  This correction accounts for the different sizes and spatial radiance distributions of blackbody and laser-sphere sources.
3. A sphere non-uniformity correction, defined as the inverse of the average relative sphere radiance of eq (27). This correction accounts for the fact that the spectroradiometer viewed primarily the center of the sphere aperture, while the silicon and ECR detectors viewed the entire aperture.

#### 5.2.4 Diffraction Effects

The theory presented above does not include all diffraction effects which constitute a potential source of error in the physical model leading to eq (30). For the spectroradiometer measurements, diffraction is implicit in the slit-scattering and spatial apparatus functions considered. Diffraction losses at the limiting aperture of the spectroradiometer were eliminated by limiting the field of view, not at the laser sphere or blackbody, but within the spectroradiometer system, so that the effects would cancel when the ratio $S_{S}^{\mathrm{BB}}/S_{S}^{\mathrm{LS}}$ in eq (31) is taken. However, for the silicon-detector and ECR measurements, there may be a small diffraction loss caused by the apertures being mounted a few millimeters in front of the detectors themselves. We performed calculations based on the treatment of Steel, De, and Bell [34] and found losses ranging from 0.003% for the silicon detector at 514 nm to 0.02% for the ECR at 647 nm. The upper limit of this range (0.02%) was included in our error analysis.

## 6. Measurements

### 6.1 Spectroradiometer Slit-Scattering Function Correction, *f*_σ_

This correction factor was calculated from eq (32) by combining the measured values of the spectroradiometer spectral responsivity *R*_s_(λ) (fig 9) and slit-scattering function σ(λ,λ′) (fig. 7) with calculated values of the Planck function *P*(λ,*T*) [eq (3)] evaluated at the IPTS-68 gold-point temperature, 1337.58 K. For these calculations we used the 0.6-mm slit-scattering functions obtained in our earlier study, because they include far-wing data. Since the slit-scattering function appears in integrals in both the numerator and denominator of f_σ_, it tends to cancel and small differences between the functions obtained previously and the current slit-scattering functions are of no consequence. The values of *f*_σ_ at the three laser wavelengths fall within the range 0.9999+0.0004.

### 6.2 Spectroradiometer Spatial Apparatus Function Correction, *f*_H_

To compute the value of *f*_H_ defined by eq (33), we used the normalized horizontal and vertical traverses of the spatial apparatus function of the spectroradiometer shown in figure 8 and the measured spatial radiance distributions of the blackbody and laser-sphere sources shown in figures 5 and 6. These distributions, of course, represent convolutions of the true distributions with the spectroradiometer point-spread function, whereas the functions needed to calculate *f*_H_ are the true distributions. In the evaluation of the integral in the denominator of eq (33) only the central 1 or 2 mm contribute significantly, and here the effect of convolution is merely a reduction of the apparent radiance by a nearly constant factor (e.g., 0.9997). Consequently, the normalized observed distribution is virtually indistinguishable from the true distribution insofar as the evaluation of the denominator in eq (34) is concerned, and no deconvolution is required. A similar remark applies to the laser-sphere integral appearing in the numerator, although in this case no deconvolution correction would have been expected anyway due to the uniformity of that distribution. The values obtained for *f*_H_ range from a minimum of 1.0002 at the end of a melt at any wavelength to a maximum of 1.0008 at the end of a freeze at 514 nm. Our final result for *f*_H_ is the average of all calculated values, *f*_H_= 1.0003+0.0006, where the uncertainty covers the dependence upon time and wavelength and includes an uncertainty for the contribution of the far wings of the spatial apparatus function, extrapolated beyond ±7 mm.

### 6.3 Laser-Sphere Nonuniformity Correction, <*ℓ*_LS_>

The integration of the measured distribution over the laser-sphere source (fig. 6) was performed at all three wavelengths used. The average relative radiance obtained was <*ℓ*_ls_> = 1.0022+0.0009, where the uncertainty is a three-sigma estimate of repeatability.

### 6.4 Geometric Extent of Absolute Detectors, *G*_D_

For the evaluation of this quantity, eq (17) was used in a simplified form in which the areas of the laser-sphere and detector apertures were used instead of their radii. The aperture dimensions were measured on a movable stage under a microscope calibrated by the Precision Engineering Division of NIST. The microscope was focused on the edge of the aperture being measured, and then the (*x*,*y*) coordinates of the stage were recorded as the microscope cross-hairs were positioned at arbitrary intervals of about 5° or 10° around the edge of the aperture. The data points recorded in this way were sufficiently numerous (about 50) that the omission of half of them by using only alternate points resulted in a totally negligible difference in the area determination. The (*x*,*y*) coordinate pairs were converted to (*r*,θ) coordinate pairs about an origin near the aperture center (chosen so that all *r* values were equal within 0.01 mm), and the areas were calculated from

$$A=\int_{0}^{2\pi }r^{2}d\theta /2\equiv \sum_{1}^{n}r_{1}^{2}\left(\theta_{i+1}-\theta_{i-1}\right)/4,$$ (34)

with θ_0_=θ*_n_* and θ_*n*+1_≡θ_1_. These aperture measurements were repeated two or three times for each aperture by each of two operators with excellent agreement. The values obtained and their re peatabilities (three standard deviations) are given in table 5.

The separation between source and detector apertures was set each morning by holding a parallel-faced gauge block of known length (150.0 mm for most measurements) between the apertures, and adjusting the detector position until both apertures were in light contact with the gauge. In calculating the geometric extents of the two detector systems, we accounted for the fact that the laser-sphere aperture had a thickness of 0.15 mm and was beveled toward the inside of the sphere by using an effective aperture separation of 150.15 mm. The uncertainty in the length of the gauge block was assessed as 0.02 mm, and the possible error due to lack of sharpness of the aperture edges was estimated as 0.01 mm.

### 6.5 Spectral Radiance Measurements

The measurement of a single blackbody spectral radiance value constituted a day’s work which was carried out in accordance with the measurement protocol described below. The measurement was performed in a time-symmetrical fashion, so that possible drifts of the spectroradiometer blackbody signals would be eliminated in part when overall averages were taken. However, no drifts were observed outside of statistical fluctuations. All laser-sphere readings were recorded relative to simultaneously taken monitor-diode readings, so that errors due to small fluctuations of laser-sphere output were minimized and the transmittance of the neutral-density filter was automatically accounted for when it was inserted in the laser beam entering the sphere.

The individual measurement steps performed were:

1. While the blackbody was heating up to the first melt of the day, the laser was turned on and the separation *d* between the laser sphere and detector apertures was set as described in section 6.4. The ECR was positioned in front of the laser sphere, and the laser power was adjusted to give readings of approximately 0.4 mW (without neutral density filter).
2. The ECR was calibrated by applying electrical substitution heating for a duration of 180 s, and then its responsivity *r*_ecr_ (V/W) was recorded as the ratio of the thermocouple MF and heating power applied, divided by the ECR correction 1.001.
3. The ECR was exposed to radiant heating for 180 s, and the relative ECR laser-sphere signal $S_{\mathrm{ECR}}^{\mathrm{LS}}$ was recorded (in V/A) as the ratio of the thermocouple EMF and the monitor-diode reading.
4. The neutral-density filter was inserted in the beam and the spectroradiometer positioned in front of the laser sphere. The central 10-nm portion of the spectroradiometer slit-scattering function was recorded by scanning the monochromator wavelength drive (514 and 633 nm) or scanning the dye laser (647 nm). The monochromator was set to the maximum of this scan, where it remained until the measurements were complete for the day. A preliminary value, ∆λ_0_, of the spectroradiometer bandwidth was calculated from eq (31) by integrating the measured slit-scattering function. This result was corrected for the omitted contribution of the far wings of the slit-scattering function as described in section 4.4.
5. Once the blackbody was in a melt (or, later, in a freeze), a cycle of three readings was taken repeatedly: the spectroradiometer readings of the blackbody and the laser sphere, and the silicon and monitor diode readings of the sphere. The spectroradiometer blackbody signal $S_{S}^{\mathrm{BB}}$ was recorded as the measured photomultiplier anode current (A). The spectroradiometer and silicon-diode laser-sphere signals, $S_{S}^{\mathrm{LS}}$ and $S_{\mathrm{Si}}^{L\;S},$ were recorded relative to the monitor-diode readings (in dimensionless units).
6. During the hour or so following a melt (or freeze) and preceding the subsequent freeze (or melt), the laser sphere was repeatedly measured with the ECR and the silicon and monitor detectors, as described in steps (3) and (5). Generally, two to four melt-freeze cyles were carried out in a day, each time repeating the measurements of step (5).
7. Following the last freeze of the day, the laser sphere was re-measured with the ECR and the silicon and monitor detectors, the spectroradiometer slit-scattering function was re-measured as in step (4), and the ECR was re-calibrated by electrical substitution as in step (2).

Table 6 gives a representative sample of the average data obtained at 647 nm on a typical day. The table also gives the values of the various other factors needed to calculate the blackbody spectral radiance from eq (30). In the example shown, the silicon-detector and ECR results are 63.319 and 63.395 Wcm^−3^sr^−1^, respectively.

### 6.6 Blackbody Spectral Radiances

The measurement sequence described in section 6.5 was performed five times at each laser wavelength. This gave 10 spectral radiance values, each, at the argon and krypton laser wavelengths, and five values at the helium-neon laser wavelength. The 25 spectral radiance values thus obtained are listed in table 7 and were treated as single measurements for data reduction purposes.

Because our experiment relies on the redundant detector scales established by the silicon diode and the ECR, we verified the mutual agreement of these scales by comparing the differences and the standard deviations of the silicon-diode and ECR values of table 7. As shown at the bottom of the table, the two scales were found to agree within experimental uncertainties.

The standard deviations of the mean listed in table 7 represent the composite random uncertainties of all of the measurements described in section 6.5. They include the random uncertainties of all detector signals, the calibration of the ECR, the wavelength setting of the spectroradiometer and measurement of its bandwidth, the setting of the aperture distance, the transmittance of the neutral density filter, as well as fluctuations of the blackbody and laser-sphere sources. Since the individual standard deviations are all on the order of 0.1%, our three-sigma estimate of the composite random uncertainty of these measurements is 0.3%.

### 6.7 Gold-Point Temperature

To find the gold-point temperature *T*_Au_ we expressed the theoretical blackbody radiance as the product of Planck’s eq (3) and the estimated blackbody emissivity, *ϵ*=0.9999, of section 4.1. We compared the average measured spectral radiances $L_{\lambda }^{\mathrm{BB}}$ given in table 7 with the values ∊ *P*(λ,*T*_Au_) obtained by substituting trial values of *T*_Au_, the laser wavelengths listed in table 3, the refractive indexes of air computed from Edlen’s formula [35], and evaluated the radiation constants, *c*_1_= *2hc*^2^ and *c*_2_=*hc/k*, in terms of the 1986 values of *h*, *k*, and *c* given by Cohen and Taylor [36], By varying *T*_Au_ until the quadrature sum of the weighted differences $\left[L_{\lambda }^{\mathrm{BB}}-\epsilon P\left(\lambda ,T_{\text{Au}}\right)\right]/\sigma_{\lambda }$ (σ_λ_ being the standard deviations of the mean given in table 7) was minimized, we found *T*_Au_= 1337.334 K. The relative standard deviation of the mean of this least-squares fit was ±0.056% (relative to radiance). The theoretical blackbody spectral radiances ∊*P*(λ,*T*_Au_) calculated for this temperature and their deviations from the average measured spectral radiances $L_{\lambda }^{\mathrm{BB}}$ are listed in table 8. These residuals exhibit a small dependence on wavelength which is unexplained, but falls within the combined uncertainty of our measurements.

### 6.8 Uncertainty

To estimate the uncertainty of this result, we added in quadrature the composite random uncertainty of the blackbody spectral radiance measurements (sec. 6.6), the uncertainty of the least-squares fit (sec. 6.7), and the various systematic uncertainties in excess of 0.01% that have been mentioned in previous sections of this paper. These uncertainties have been collected in table 9, where all of them are given as estimated upper limits of systematic errors or three standard deviations. Their summation in quadrature gives a total uncertainty (relative to radiance) of 0.45%. Hence, the uncertainty of the measured gold-point temperature was estimated from eq (10) as ±0.34 K (at wavelength 600 nm).

## 7. Result

Based on the above, the value

$$T_{\text{Au}}=\left(1337.33\pm 0.34\right)K$$ (35)

is quoted as the final result of our measurement of the gold-point temperature.^2^ This result is in good quantitative agreement with the measurements described in section 3, above. Our experiment represents the only direct spectroradiometric determination of the gold-point temperature which has been reported so far and, therefore, provides an independent confirmation of these earlier measurements. The value quoted in eq (34) was submitted to the Consultative Commmittee on Thermometry as an official NIST contribution to the ITS-90 and, as noted in section 1, was incorporated into the new scale.

## Figures and Tables

**Table 1:** Quoted uncertainties (3σ) of current NIST measurement services for radiation thermometry, radiometry, and photometry and estimated changes in reported values resulting from a −0.25 **K** reassignment of the gold-point temperature

Quantity	Uncertainty	Change of value
Radiance temperature [4]		
800 °C	±0.5 °C	−0.2 °C
1100	0.6	−0.2
1400	0.8	−0.4
1800	1.3	−0.6
2300	2.0	−0.9
Spectral radiance [3]		
250 nm	±1.6%	−0.8%
350	1.2	−0.6
650	0.6	−0.3
900	0.5	−0.2
1600	0.4	−0.1
2400	0.4	−0.1
Spectral irradiance [5]		
250 nm	±2.3%	−0.8%
350	1.4	−0.6
650	1.0	−0.3
900	1.3	−0.2
1600	1.9	−0.1
2400	6.5	−0.1
Luminous intensity [6]		
2856 K	±1.0%	−0.4%
Luminous flux [6]		
2856 K	±1.4%	−0.4%

**Table 2:** Results and uncertainties (3σ) of gold-point measurements performed since 1971

Authors	Reference Temperature	Result (K)
Blevin and Brown [11]	None	1337.27±0.4
Bonhoure [13], [17]	Antimony	1337.20±0.5
Andrews and Gu [14], [2]	Antimony	1337.33±0.3
Tischler and Rebagliati [17]	Antimony/Silver	1337.22±0.5

**Table 3:** Laser wavelength used in this experiment [19]

Laser	Transition	Air wavelength (µm)
Ar II	$3p^{4}{(}^{3}P)4p{(}^{4}D_{5/2}^{0})\to 3p^{4}{(}^{3}P)4s{(}^{2}P_{3/2})$	0.514533
HeNe	$2p^{5}{(}^{2}P_{1/2})5s{\left[1/2\right]}_{1}^{0}\to 2p^{5}{(}^{2}P_{1/2})3p{\left[3/2\right]}_{1}$	0.63281646
Kr II	4*p*^4^(^3^P)5*p*(^4^P_5/2_)→4*p*^4^(^3^P)5*s*(^3^P_3/2_)	0.647100

**Table 4:** Correction factors for ECR spectral responsivity and estimated uncertainties (3σ)

	Factor	Uncertainty (%)
Electrical lead heating	1.0000	0.06
Radiant case heating	1.0000	0.05
Reflection and emission loss	1.0001	0.10
Convection-conduction loss	1.0010	0.10
Non-uniformity of response	0.9998	0.05
Infrared response	1.0001	0.01
Overall correction	1.0010	0.17

**Table 5:** Aperture dimensions and distances

Laser-sphere aperture area	(2.5523+0.0013) cm^2^
Laser-sphere aperture thickness	(0.015±0.001) cm
Si-detector aperture area	(0.50021 ±0.00035) cm^2^
ECR aperture area	(0.2835±0.0004) cm
Aperture distance	(15.000±0.002) cm
Geometric Extent, *G*_si_	(5.63843±0.00052) 10^−3^ cm^2^sr
Geometric Extent, *G*_ecr_	(3.19662±0.00049) 10^−3^ cm^2^sr

**Table 6:** Representative data set obtained at 647.1 nm

ECR responsivity, *R*_ECR_	0.267244 V/W
Relative ECR signal, $S_{\mathrm{ECR}}^{\mathrm{LS}}$	0.19412 V/W
Spectroradiometer bandwidth (including far wings), ∆λ	2.5273×10^−7^cm
Spectroradiometer blackbody signal, $S_{S}^{\mathrm{BB}}$	3.17858×10^−8^ A
Relative spectroradiometer laser-sphere signal, $S_{S}^{\mathrm{LS}}$	0.449073
Relative silicon-diode signal, $S_{\mathrm{Si}}^{\mathrm{LS}}$	0.599112
Silicon-diode responsivity, *R*_si_	0.468170 A/W
Silicon-diode geometric extent, *G*_Si_	5.63843×l0^−3^cm^2^sr
ECR geometric extent, *G*_ECR_	3.19662×l0^−3^cm^2^sr
Spectroradiometer slit-scattering correction, *fσ*	0.9999
Spectroradiometer apparatus-function correction, *f*_H_	1.0003
Laser-sphere nonuniformity correction, <*ℓ*_LS_>	1.0022
Blackbody spectral radiance (Si), $L_{\lambda }^{\mathrm{BB}}$	63.3186 Wcm^−3^sr^−1^
Blackbody spectral radiance (ECR), $L_{\lambda }^{\mathrm{BB}}$	63.3953 Wcm^−3^sr^−1^

**Table 7:** Measured blackbody spectral radiances (Wcm^−3^sr^−1^) and random uncertainties (1*σ*)

	514.5 nm		632.9 nm	647.1 nm	
	Si	ECR	Si	Si	ECR
	2.7468	2.7523	48.883	63.482	63.484
	2.7576	2.7570	48.621	63.319	63.395
	2.7656	2.7612	48.761	63.192	63.178
	2.7650	2.7605	48.659	63.396	63.379
	2.7653	2.7616	48.696	63.477	63.231
Mean	2.7601	2.7586	48.724	63.373	63.333
Std dev of mean	0.0036	0.0018	0.046	0.054	0.056
	(0.13%)	(0.07%)	(0.09%)	(0.09%)	(0.09%)
Difference	0.0015			0.040	
	(0.05%)			(0.06%)	

**Table 8:** Residuals of least-squares fit for *T*_Au_= 1337.334 K

Wavelength (nm)	Calculated radiance (Wcm^−3^sr^−1^)	Residuals	
		Si	ECR
514.5	2.7561	0.0040	0.0025
632.9	48.736	−0.12	
647.1	63.433	−0.060	−0.100

**Table 9:** Estimated uncertainties (3σ) of gold-point measurement (see sections cited in parentheses for details)

Quality	Uncertainty
Composite random uncertainty (6.6)	0.30%
Least-squares fit (6.7)	0.17%
Blackbody emissivity (4.2)	0.01%
Polarization (4.3)	0.02%
Far-wing bandwidth correction (4.4)	0.04%
Detector responsivity [silicon (4.5) or ECR (4.6)]	0.20%
Detector linearity (4.8)	0.10%
Diffraction (5.2.4)	0.02%
Slit-scattering function correction *f*_σ_ (6.1)	0.06%
Apparatus function correction *f*_H_ (6.2)	0.03%
Sphere non-uniformity correction <*ℓ*_LS_> (6.3)	0.09%
Geometric extent, *G*_D_ (6.4)	0.15%
Total radiance uncertainty (added in quadrature)	0.45%
Gold-point temperature uncertainty [from eq (10), at 600 nm]	0.34 K

## Footnotes

1 Certain commercial equipment, instruments, or materials are identified in this paper to specify adequately the experimental procedure. Such identification does not imply recommendation or endorsement by the National Institute of Standards and Technology, nor does it imply that the materials or equipment identified are necessarily the best available for the purpose.

2 This value differs from a preliminary value which was reported at an IMEKO Photon-Detector Committee meeting (Braunschweig 1987). The preliminary value was based on an incomplete analysis of the raw data reported in the present paper, and was in error because the refractive index of air (about 1.00027) had not been included in Planck’s radiation formula.
